# Symptomatic Acute Hepatitis C in Egypt: Diagnosis, Spontaneous Viral Clearance, and Delayed Treatment with 12 Weeks of Pegylated Interferon Alfa-2a

**DOI:** 10.1371/journal.pone.0004085

**Published:** 2008-12-30

**Authors:** Noha Sharaf Eldin, Soheir Ismail, Hala Mansour, Claire Rekacewicz, Moustafa El-Houssinie, Sherif El-Kafrawy, Saeed El Aidi, Mohamed Abdel-Hamid, Gamal Esmat, Stanislas Pol, Arnaud Fontanet, Mostafa K. Mohamed

**Affiliations:** 1 Department of Community Medicine, Ain Shams University, Cairo, Egypt; 2 Hepatology, National Hepatology and Tropical Medicine Research Institute, Cairo, Egypt; 3 Pharmacy, National Hepatology and Tropical Medicine Research Institute, Cairo, Egypt; 4 Emerging Diseases Epidemiology Unit, Institut Pasteur, Paris, France; 5 PCR Unit, National Liver Institute, Menoufia University, Menoufia, Egypt; 6 Imbaba Fever Hospital, Cairo, Egypt; 7 Microbiology, Faculty of Medicine, Minia University, Minia, Egypt; 8 Department of Tropical Medicine and Hepatology, Faculty of Medicine, Cairo University, Cairo, Egypt; 9 Assistance Publique des Hôpitaux de Paris, Hôpital Cochin, Unité d'Hépatologie, Paris, France; Yale University, United States of America

## Abstract

**Background and Objectives:**

The aim of this study was to estimate the proportion of spontaneous viral clearance (SVC) after symptomatic acute hepatitis C and to evaluate the efficacy of 12 weeks of pegylated interferon alfa-2a in patients who did not clear the virus spontaneously.

**Methods:**

Patients with symptomatic acute hepatitis C were recruited from two “fever hospitals” in Cairo, Egypt. Patients still viremic three months after the onset of symptoms were considered for treatment with 12 weeks of pegylated interferon alfa-2a (180 µg/week).

**Results:**

Between May 2002 and February 2006, 2243 adult patients with acute hepatitis were enrolled in the study. The SVC rate among 117 patients with acute hepatitis C was 33.8% (95%CI [25.9%–43.2%]) at three months and 41.5% (95%CI [33.0%–51.2%]) at six months. The sustained virological response (SVR) rate among the 17 patients who started treatment 4–6 months after onset of symptoms was 15/17 = 88.2% (95%CI [63.6%–98.5%]).

**Conclusion:**

Spontaneous viral clearance was high (41.5% six months after the onset of symptoms) in this population with symptomatic acute hepatitis C. Allowing time for spontaneous clearance should be considered before treatment is initiated for symptomatic acute hepatitis C.

## Introduction

Management of acute hepatitis C is a complex issue. While studies have shown that treatment during the acute phase can achieve high (72%–98%) success rates [1]–[9], the optimal regimen and timing of treatment are still a matter of debate [10]–[13]. One crucial issue that remains to be resolved is whether physicians should treat all patients diagnosed with acute hepatitis C, or should wait and treat only those who failed to clear the virus in the first few months after infection. Among recently published studies (2006–7) [5]–[9], the trend has been to treat early (i.e., within three months of infection or one month of onset of symptoms), and with simplified regimens (12 or 24 weeks of monotherapy with pegylated interferon). In these studies, intravenous drug use and occupational exposure were the main risk factors for infection, and the majority of patients were a- or pauci-symptomatic, except for the German HEP-NET study, where recruitment was more diverse and patients with jaundice represented 62% of all cases [6].

In Egypt, the epidemiological situation differs from that of Western countries. HCV prevalence is very high (estimated among adults at 10 and 20% in urban and rural areas respectively) [14]. The origin of the epidemic has been attributed to mass campaigns of parenteral anti-schistosomiasis treatment in rural areas in the 1960s–70s. Since the virus has continued to spread, mainly through intravenous injections and other medical procedures [15], acute hepatitis C is commonly diagnosed among patients presenting with jaundice [16]

Among these patients, waiting for spontaneous clearance and treating only those still viremic three months after the onset of symptoms makes sense for two reasons: the higher rate of spontaneous clearance expected from patients with symptomatic compared to asymptomatic forms of acute hepatitis C (estimated at 31% versus 18%, respectively, 17); and the cost savings incurred by not treating all patients, an important consideration in countries with limited resources, including Egypt. In this paper, we present the results of the follow-up of 117 patients diagnosed with symptomatic acute hepatitis C in Cairo. Patients who failed to clear the virus three months after the onset of symptoms were considered for treatment with 12 weeks of pegylated interferon alfa-2a.

## Methods

### Study population and questionnaire

The recruitment of patients with symptomatic acute hepatitis C has already been described in our previous report of a pilot study performed in 2002 in Cairo [16]. Adjustments to the protocol were made at the end of the pilot study, and the main components of the study are presented here. Patients were recruited from two “fever hospitals” (Abbassia and Imbaba Fever Hospitals), which are public hospitals specialized in infectious diseases offering care at moderate cost to the disadvantaged populations of Cairo. Inclusion criteria were age above 18 years, symptoms (fever or jaundice) lasting less than 21 days, and elevated serum alanine aminotransferase (ALT) ≥3 times the upper limit of normal (ULN = 40 IU/L).

All patients were interviewed by trained medical doctors, using a questionnaire on socio-demographic characteristics and risk factors for HCV infection in the past six months. Risk factors were categorized as high risk and low risk exposures based on the magnitude of the associations with HCV transmission documented in previous studies [15], [16]. High risk exposures included surgery, blood transfusion, hemodialysis, biopsy, endoscopy, Caesarean section, episiotomy, uterine curettage, injection, infusion, catheter, sclerotherapy of varicose veins, and dental care. Low risk exposures included acupuncture, shaving at barber, tattooing, pedicure, manicure, and circumcision. Also assessed was exposure to other potential causes of hepatitis, including drugs, pesticides, and other chemicals known for their hepatotoxicity.

### Laboratory methods

A 10 ml venous blood sample was collected for liver functions [ALT, aspartate aminotransferase (AST), total and indirect bilirubin, alkaline phosphatase], and exclusion of acute hepatitis A and B by serological analysis [IgM anti-hepatitis A virus (HAV) (HAVAB®, M EIA, Abbott Laboratories, Diagnostics Division, Abbott Park, IL); IgM anti-hepatitis B virus (HBV) core (CORZYME®, M rDNA, Abbott Laboratories, Diagnostics Division); hepatitis B surface antigen (AUSZYME MONOCLONAL®, third generation EIA, Abbott laboratories, Diagnostics Division, Abbott Park, IL)]. In patients with non-A non-B hepatitis, anti-HCV antibodies were assessed serologically (INNOTEST® HCV Ab IV, Innogenetics, Ghent, Belgium), and HCV RNA Extraction was performed using QIAmp Viral RNA Kit (QIAGEN, Santa Clarita, U.S.A.) according to manufacturer's recommendations. The Reverse Transcription and Polymerase Chain Reaction (RT-PCR) was done according to Abdel-Hamid et al, 1997 [18] with some modifications to increase the sensitivity of the assay (50 IU/mL). The RT and first round of PCR amplification were performed in a single step as follows: the RT-PCR master mix consisted of 1X Taq buffer with 1.5 mM MgCl2 (Roche Molecular Biochemicals), 0.2 mM dNTP (Promega), 20 pmol of each primer (P1 and P2), 20 U of Human Placental Ribonuclease Inhibitor (RNasin, Promega), 10 U of AMV reverse transcriptase (Promega), and 2.5 U of Taq DNA polymerase (Roche Molecular Biochemicals). The master mix was added to 55 µl of RNA from each sample, and the tubes were subjected to the following thermal cycles: 42°C for 30 minutes (one cycle) for RT, 35 cycles at 94°C for denaturation for 1 minute, 50°C annealing for 1 minute, and 72°C extension for 1 minute and a final extension step at 72°C for 10 minutes (one cycle). Nested PCR was conducted with 10 µl of the first PCR reaction added to 90 µl of the master mix, consisting of the same reagents used in the first PCR expect for using P-3 and P-4 and without adding RT or RNasin. Thermo cycling for 35 cycles was performed as in the first PCR but without the initial RT step. The outer (P-1, P-2) and inner primers (P-3, P-4) for the nested PCR reaction were derived from the highly conserved 5′ untranslated region (5′UTR) of the HCV genome. PCR results were visualized by electrophoresis using ethidium bromide in a 3% agarose gel.

In patients with positive HCV antibodies and RNA, exacerbation of chronic hepatitis C by other infectious agents was ruled out using reverse transcriptase PCR for HEV-RNA (in house assay using ORF1 and ORF2 primers) and serological testing [anti Epstein-Barr virus (EBV) IgM (ETI-EBV-M reverse P001605, Dia Sorin, Vercelle, Italy), anti-cytomegalovirus (CMV) IgM (AXSYM® system-CMV-IgM, Abbott Laboratories, Wiesbaden, Delknheim, Germany), and anti-Toxoplasma IgM (AXSYM® system-Toxo-IgM, Abbott Laboratories, Wiesbaden, Delknheim, Germany)]. All tests were performed at the Viral Reference Hepatology Laboratory (VHRL).

### Definitions of acute hepatitis C

The diagnosis of acute hepatitis C was based on epidemiological findings, clinical examination, and laboratory test results. Accordingly, patients were categorized as *1) Definite acute hepatitis C* if negative anti-HCV antibody and positive HCV RNA; *2) Probable acute hepatitis C* if positive anti-HCV antibody and HCV RNA associated with ALT≥10 times the ULN, history of high risk exposure 1 to 3 months prior to diagnosis, negative serology/PCR (see above) for other infectious agents (HAV, HBV, HEV, EBV, CMV, toxoplasmosis), no history of drugs or pesticides exposure, and no other cause of hepatitis (e.g., autoimmune hepatitis, ischemic liver injury, Wilson disease).

### Follow-up

All patients with acute hepatitis C were offered follow-up at the National Hepatology and Tropical Medicine Research Institute (NHTMRI) in Cairo. Visits were scheduled for clinical and biochemical assessment at 2, 3, 6, 12, 18 and 24 months, calculated from onset of symptoms. Antiviral treatment was not offered immediately in order to allow for spontaneous clearance of the virus; however, non-specific symptomatic treatment, such as antiemetics for nausea and vomiting, antipyretics for fever and analgesics for headache, were offered to all patients. Patients who were still HCV RNA positive three months after the onset of symptoms were screened for treatment eligibility. Exclusion criteria for treatment were age <18 years or >65 years; poorly controlled diabetes; thyroid disease (TSH outside the normal range); autoimmune diseases; alcoholism; liver cirrhosis; hepatocellular carcinoma; psychiatric disease such as history of severe depression, psychosis, suicidal ideas; epilepsy; non-stabilized medical or surgical condition; hemoglobin<11 g/dl, leucocytes <3000/µL, polynuclear neutrophils <1500/µL, platelets <100 000/µL, and blood creatinin >150 µmol/L. For females: pregnancy or breastfeeding were contra-indications (an effective contraception was requested during the treatment period).

Treatment with free pegylated interferon [PEG-IFNα-2a (PEGASYS®, Roche)] (180 µg/week) for 12 weeks was started 4–6 months after onset of symptoms in those still positive at that date, provided there were no contraindications. Injections were provided at the NHTMRI clinic under medical supervision. In patients still HCV RNA positive after 12 weeks of treatment, treatment was extended for another 12 weeks. Patients were seen weekly during the treatment period, and every six weeks afterwards until week 36 (end of follow-up for those treated for 12 weeks), or week 48 (end of follow-up for those treated for 24 weeks). The dosage of PEG-IFN α-2a was reduced for two weeks to 90 µg/week if neutrophil or platelet counts fell below 750/µL and 50,000/µL, respectively. After the 2 weeks, dose was raised back to 180 µg/week if neutrophil and platelet counts went above 750/µL and 50,000/µL, respectively. Treatment was discontinued if neutrophil or platelet counts fell below 500/µL and 25000/µL, respectively, or when serum hemoglobin concentrations decreased below 8.5 g/dL. Doctors' judgment was the basis for dose reduction and discontinuation of PEG-IFN α-2a if other commonly reported adverse events (such as flu-like symptoms and emotional side effects) were reported. The primary endpoint was sustained virological response (SVR), defined as negative serum HCV RNA 24 weeks after the end of treatment. Secondary endpoints were the absence of detectable levels of serum HCV RNA at the end of treatment and the normalization of ALT levels. Viral load was determined using the Cobas Amplicor HCV Monitor test, v 2.0 (Roche Diagnostic Systems).

### Ethics

All patients with acute hepatitis consulting or hospitalized in the two participating hospitals were included in the study after providing a written informed consent. A separate informed consent was required for the patients who were treated with pegylated interferon. The entire study protocol has obtained clearance from the Ministry of Health and Population in Egypt, and the Institutional Review Board for Human Subject Research at NHTMRI. The 12-week pegylated interferon trial protocol for patients with acute hepatitis C has been registered under the following number: NCT00158522 (ANRS1213).

### Statistical Analysis

Spontaneous viral clearance (SVC) was defined as no HCV RNA in serum, in the absence of treatment, for two consecutive HCV PCR tests during follow-up. Patients treated with pegylated interferon were censored at the time of treatment initiation. Date of clearance was chosen as the midpoint between the last positive PCR and the first negative PCR defining clearance. The SVC rate was estimated using Kaplan-Meier estimates, with time 0 equivalent to onset of symptoms. A Cox proportional hazards model was used to identify factors associated with SVC. Factors with p values less than 0.25 in univariate analysis were tested in the multivariate model, and p values less than 0.05 were considered significant. Statistical analyses were performed using Stata 8 statistical software (Stata Corporation, College Station, Texas, USA).

## Results

### Diagnosis of acute hepatitis C (Figure 1)

Between May 2002 and February 2006, 2243 consecutive adult patients with acute hepatitis were recruited from Abbassia and Imbaba Fever Hospitals. Of these, 647 (28.8%) were diagnosed with acute hepatitis A, 609 (27.2%) were diagnosed with acute hepatitis B, and 401 (17.9%) tested positive for HCV RNA by PCR. Of the patients positive for HCV RNA, those with negative HCV antibodies (n = 79; 3.5% of the initial 2243 patients) were considered as definite acute hepatitis C. Of the 322 with positive HCV antibodies, 114 had ALT levels lower than 10 times ULN, and were therefore no longer considered potential acute hepatitis C cases. Full assessment to determine final status (serologies/PCR; see Patients and methods) was performed for the remaining 208 patients, and 81 were diagnosed with probable acute hepatitis C. Therefore, the overall proportion [95% CI] of acute hepatitis C among all acute hepatitis adult patients was 160 (i.e., 79+81)/2243 = 7.1% [6.1%–8.3%].

**Figure 1:**
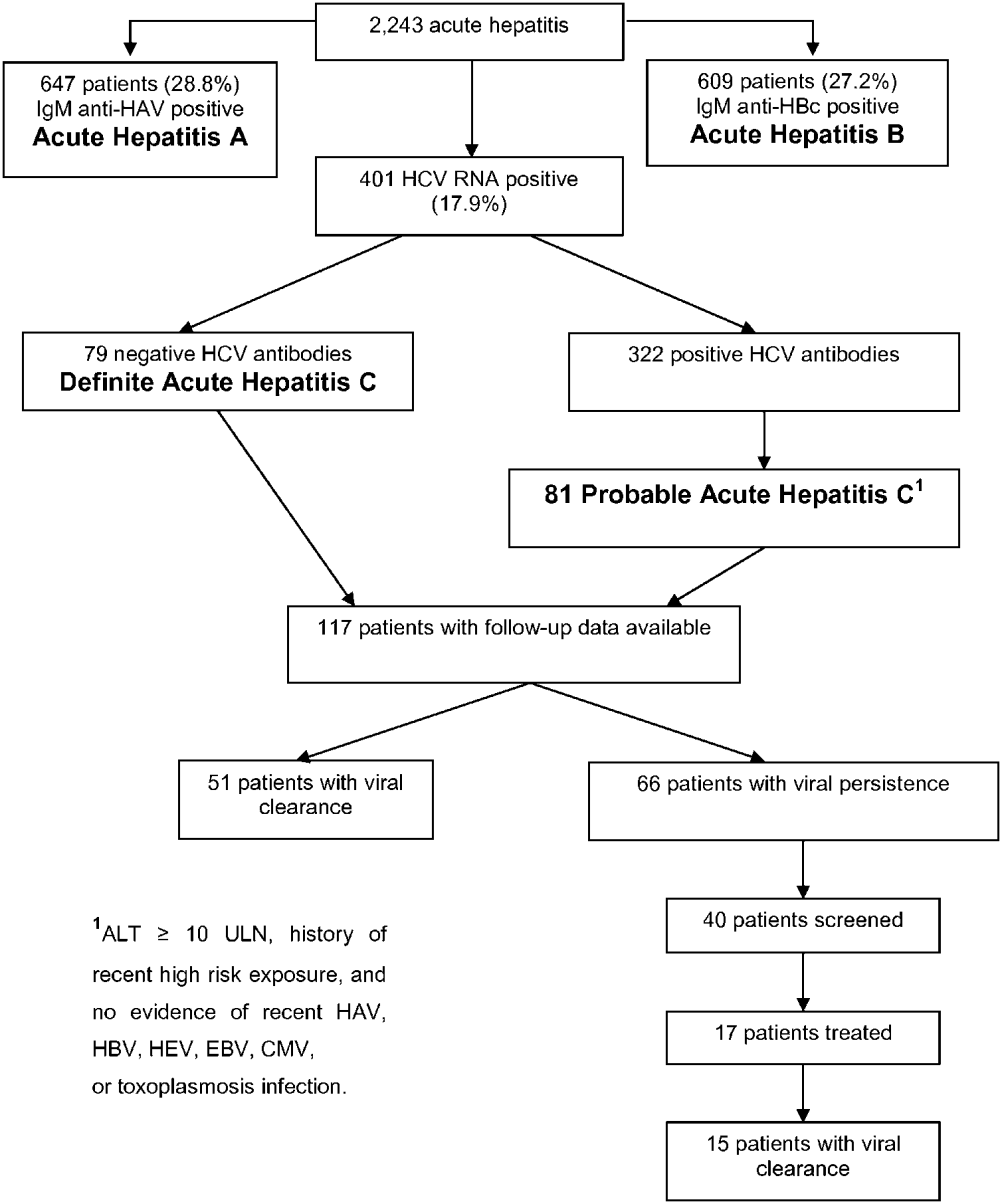
Diagnosis and follow-up of acute hepatitis C among 2243 patients with acute hepatitis, 2002–6, Cairo.

### Follow-up

Follow-up data were available for 117 (73.1%) of the 160 patients with acute hepatitis C. Table 1 displays the socio-demographic, clinical, and biological characteristics of the 160 acute hepatitis C patients. There was no difference in these characteristics among acute hepatitis C cases followed-up and those who were not, except for ALT level. Patients with probable acute hepatitis C were all in the group with follow-up since ascertainment of cases required follow-up to perform additional serological and PCR investigations (see Patients and Methods). As a result, patients with follow-up had higher median ALT level when compared to those without follow-up since the definition of probable acute hepatitis C included ALT >10 times the ULN. The following is a description of the patients with follow up. The mean age of patients was 34.6 years and 59% were males. Due to the location of the fever hospitals in Cairo, the majority of patients belonged to an urban population (78.1%). Forty percent were illiterate, reflecting the low socio-economic status of patients attending the fever hospitals. Nearly all patients presented with jaundice (96.6%) and one third presented with fever (35.0%). Median ALT level was above 15 times the upper limit of normal (ULN). Potential sources of HCV infection were medical (n = 77; 65.8%), dental (n = 5; 4.3%), intravenous drug use (n = 9; 7.7%), low risk exposure (n = 19; 16.2%), and unknown (n = 7; 6.0%). Of the potential medical sources of infection, surgery was the most commonly reported (40/77 = 51.9%), and provided a precise putative date of infection. The mean time from infection to onset of symptoms was 1.6 months among these patients.

**Table 1:** Demographic, clinical, and biological characteristics of acute hepatitis C patients with and without follow-up, Cairo, 2002–6 (n = 160).

Characteristics		Completed Study (n = 117)	Lost to Follow up (n = 43)	P-value
*Age (in years)*	≤35 years	69 (59.0)	29 (67.4)	0.33
	>35 years	48 (41.0)	14 (32.6)	
*Gender*	Female	48 (41.0)	19 (44.2)	0.75
	Male	69 (59.0)	24 (55.8)	
*Residency*	Urban	89 (78.1)	35 (81.4)	0.65
	Rural	25 (21.9 )	8 (18.6)	
	Missing	3	0	
*Marital Status*	Ever married	86 (73.5)	26 (60.5)	0.14
	Never married	31 (26.5)	17 (39.5)	
*Education*	Illiterate	46 (40.4)	17 (39.5)	0.14
	Read and write	39 (34.2 )	9 (21.0)	
	Formal education	29 (25.4)	17 (39.5)	
	Missing	3	0	
*Occupation*	Skilled labour	13 (11.3)	6 (14.0)	0.21
	Unskilled labour	61 (53.0 )	16 (37.2)	
	Not working	41 (35.7)	21 (48.8)	
	Missing	2	0	
*Fever*	Yes	41 (35.0)	16 (37.2)	0.91
	No	76 (65.0)	27 (62.8)	
*Jaundice*	Yes	113 (96.6)	42 (97.7)	0.81
	No	4 (3.4)	1 (2.30)	
*Laboratory Data*	Median (IQR) ALT (IU/L)	684 (515–904)	510 (299–650)	0.001^1^
	Median (IQR) total bilirubin (mg/dl)	7.2 (3.8–10.9)^2^	9.1 (4.7–11.8)	0.29

1 Patients with probable acute hepatitis C were all in the group with follow-up since ascertainment of cases required follow-up to perform additional serological and PCR investigations (see Patients and Methods). As a result, patients with follow-up had higher median ALT level when compared to those without follow-up since the definition of probable acute hepatitis C included ALT>10 times the ULN.

2 Two missing values for bilirubin

**Figure 2:**
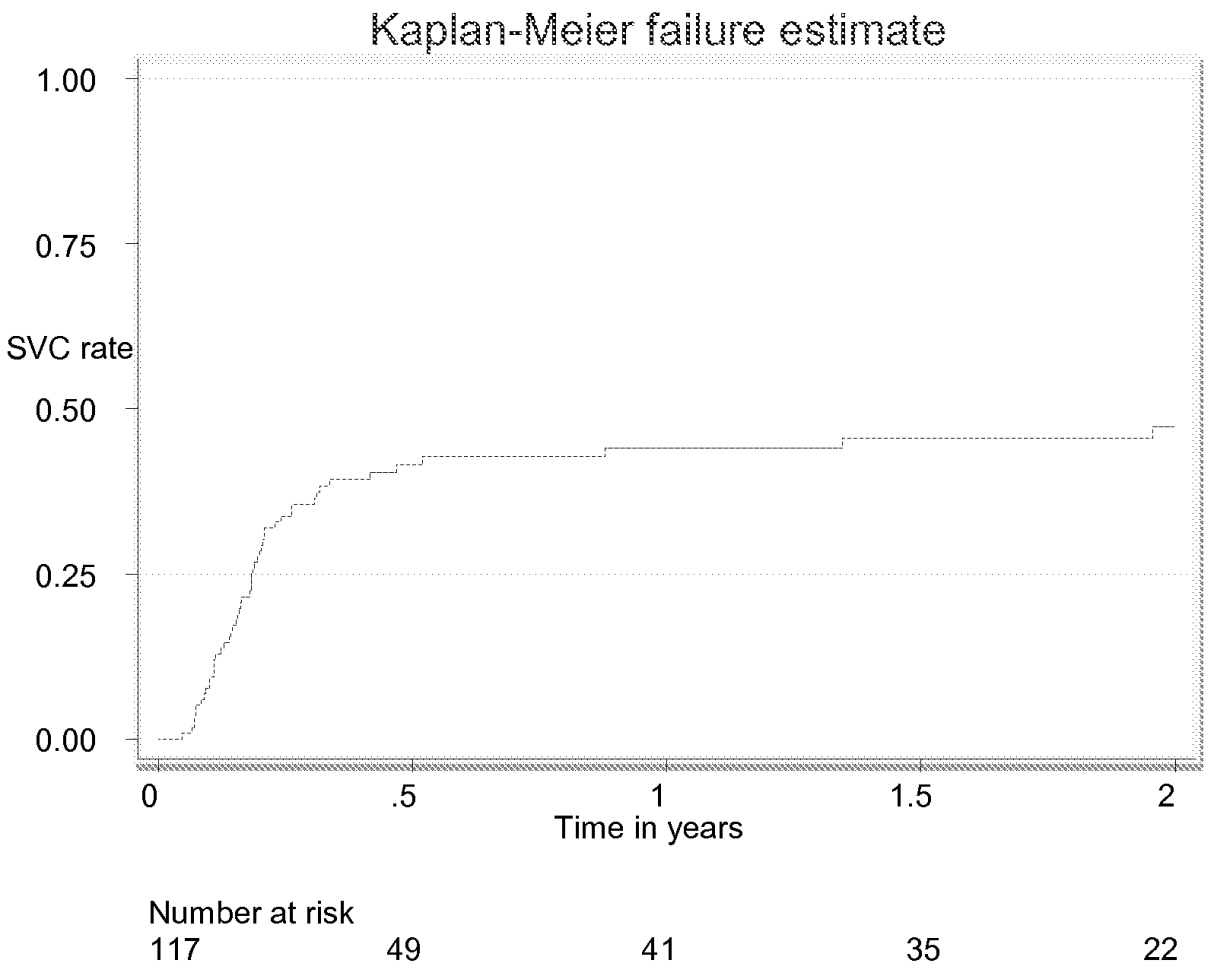
Kaplan-Meier estimate of the SVC rate among 117 patients with acute hepatitis C, 2002–6, Cairo.

In total, 51 patients spontaneously cleared the virus, with the following Kaplan-Meier estimates (95% CI) at specific points during the follow-up period: 33.8% (25.9%–43.2%) at 3 months, 41.5% (33.0%–51.2%) at 6 months, 44.0% (35.2%–53.9%) at 12 months, 45.5% (36.5%–55.6%) at 18 months, and 47.3% (38.0%–57.6%) at 24 months (Figure 2). For those who cleared the virus spontaneously, median (IQR) time from onset of symptoms to HCV RNA clearance was 66 (40–88) days, and 80.4% had cleared the virus by day 96. Only 4 patients cleared the virus more than six months after the onset of symptoms (at days 190, 321, 492, and 715). None of the studied risk factors (including age, gender, clinical and laboratory findings, and source of infection) were associated with SVC in univariate analysis (Table 2). In some patients, atypical patterns of viremia were observed. Eighteen of the patients with viral persistence had one negative result during their period of follow up (8/18 before 3 months, 17/18 before 6 months). Finally, there were six patients who were considered as relapsers, i.e., they had two consecutive negative PCR test results, before becoming positive again. None of them were intravenous drug users.

**Table 2:** Factors associated with spontaneous viral clearance in acute hepatitis C patients (n = 117).

Characteristics		SVC (n = 51)	Person-months	Crude HR (95%CI)	P-value
*Age (in years)*	≤35	31	652	1.00	0.47
	>35	20	542	0.81 (0.46–1.43)	
*Gender*	Female	21	527	1.00	0.80
	Male	30	667	1.07 (0.61–1.88)	
*Fever*	No	33	767	1	0.96
	Yes	18	427	0.99 (0.55–1.75 )	
*Jaundice*	No	1	28	1	0.96
	Yes	50	1166	1.05 (0.14–7.63)	
*Baseline ALT level*	≤median^1^	23	616	1	0.26
	>median^1^	28	578	1.37 (0.79–2.39)	
*Baseline bilirubin level*	≤median^1^	25	546	1	0.81
	>median^1^	25	647	0.93 (0.54–1.63)	
*Source of infection* 2	Medical	31	804	1.00	0.69
	Dental	3	75	1.51 (0.46–4.96)	
	IVDU	4	90	1.22 (0.43–3.46)	
	Low risk	11	168	1.61 (0.81–3.22)	
	Unknown	2	57	0.79 (0.19–3.31)	

1 Median ALT was 684 IU/L; median bilirubin was 7.2 mg/dl (two missing values for bilirubin).

2 Medical procedures included surgery, blood transfusion, hemodialysis, biopsy, endoscopy, Caesarean section, episiotomy, uterine curettage, injection, infusion, catheter, sclerotherapy of varicose veins. Low risk exposures included acupuncture, shaving at barber, tattooing, pedicure, manicure, and circumcision.

### Treatment with 12-week pegylated interferon alfa-2a

From the 77 patients still HCV RNA positive three months after the onset of symptoms, 17 were treated with pegylated interferon. Sixty patients were not treated for the following reasons: 16 had negative HCV RNA by PCR during the screening period (7 who definitely cleared the virus and 9 who turned positive again during follow-up), 14 had medical contra-indications to treatment, 20 did not show up at the pre-treatment screening visit but re-appeared at subsequent follow-up visits more than six months after the onset of symptoms, 6 were definitely lost to follow-up, and 4 refused treatment. Seventeen patients started treatment with pegylated interferon. Their baseline characteristics are summarized in Table 3. All patients received all weekly injections they were entitled to. No adverse event requiring hospitalization or dose reduction was observed except for one patient whose dose was reduced after 5 weeks of treatment as a result of neutropenia (neutrophils = 500/µL). Dose was reduced for two weeks and returned back to 180 µg/week in the eighth week (neutrophils = 1000/µL). This patient eventually achieved SVR. By the end of the 12-week treatment, 15/17 (88.2%) had cleared the virus. Treatment was continued for another 12 weeks for the 2 patients who tested HCV RNA positive at 12 weeks; one of them remained HCV RNA positive for the rest of the follow-up while the other cleared the virus between week 12 and week 24. However, one patient who had cleared the virus by week 12 became HCV RNA positive again by week 24. The overall SVR rate was 15/17 = 88.2% (95%CI = 63.5%–98.5%). By week 12, ALT were below the ULN (40 IU/L) in 12/17 (70.6%) patients (of note, the highest value was 52 IU/L, still quite close to the ULN). By 24 weeks after the end of treatment, ALT were normal in all except the two viremic patients (65 and 305 IU/L, respectively). Because of low study numbers, no attempt was made to look for associations between baseline characteristics and SVR.

**Table 3:** Characteristics of the 17 patients who underwent treatment with pegylated interferon

Characteristics	
Age (in years), median (IQR)	31 (27–38)
Males, n (%)	12 (70.6)
Weight (in kilos) , median (IQR)	85 (68–93)
Duration between onset of symptoms and treatment (in months), median (IQR)	5.2 (4.3–6.2)
HCV genotype, n	
4a	11
1	1
untypable	1
missing	4
ALT at treatment initiation (in IU/L), median (IQR)	85 (36–155)
Viral load at treatment initiation (in IU/mL), median (IQR)	74400 (7900, 444000)

## Discussion

Between May 2002 and February 2006, approximately 7% of adult patients presenting with acute hepatitis in two hospitals of Cairo had acute hepatitis C. In absolute numbers, around 3 cases per month were diagnosed in these two hospitals during the study period. This figure contrasts with the rarity of acute hepatitis C diagnoses in Western countries. Most patients had recent exposure to invasive medical procedures, half of which were surgical cases, suggesting the need for strict preventive measures to control this source of infection.

In the ensuing cohort of 117 patients with symptomatic acute hepatitis C, 41% had spontaneously cleared the virus six months after onset of symptoms. This figure, found in a patient group infected primarily with HCV genotype 4, is similar to the 41% estimate obtained from a cohort of patients with symptomatic community-acquired acute hepatitis C in Italy whose infecting genotypes were: 1 (42%), 2 (20%), 3 (16%), 4 (2%), and unknown (20%) [19]. Clearance after six months was a rare event in the Egyptian cohort, occurring in just four patients, bringing the overall spontaneous clearance rate estimate to 47.3% after two years. Unfortunately, as is the case for the study performed in Italy, no factors associated with SVC, which could serve as early predictors during screening for treatment, could be identified.

One of the crucial issues in acute hepatitis C management is when to treat. While randomized trials comparing different timing of treatment initiation may be the most rigorous approach to the issue, more than 200 patients in each arm would be required to have 80% power to show a 10% difference (e.g., 80% versus 90% SVR rates) between two strategies. Such sample sizes will be difficult to achieve for a rare disease like acute hepatitis C. Descriptive studies like this one may therefore be useful in making educated guesses for patient management until the results of randomized trials are available. In this study of symptomatic acute hepatitis C, 80% of all SVC took place within three months after onset of symptoms (or five months after infection taking into account the two months average duration between infection and symptoms). Thus, waiting for three months after onset of symptoms to treat may be necessary in symptomatic acute hepatitis C to allow SVC to take place. The question arises of whether waiting so long after infection to provide treatment might compromise the response of the patients who will ultimately need it. A study in which treatment with interferon was initiated one year after the onset of symptoms achieved a disappointing 40% SVR rate [3]. However, waiting one year may have been excessive, as suggested by the better results obtained in two other studies with treatment starting at least three months after the onset of symptoms. One, in which interferon-alfa was used alone or in combination with ribavirin for a mean duration of 38 weeks, achieved a 78% SVR rate among 18 patients who initiated treatment in median 7.4 (IQR: 5.5–11.6) months after the onset of symptoms [2]; another, in which 24 weeks of pegylated interferon alfa-2b were used, achieved a 94% SVR rate in 16 patients who initiated treatment after 12 weeks from onset of disease [4]. The present study, the first one offering delayed treatment with only 12 weeks of pegylated interferon, compares well with these two previous studies (SVR was 88.2% in 17 patients treated in median 5.2 months after the onset of symptoms; treatment duration was extended to 24 weeks in two patients). There is currently no evidence, in the absence of HIV co-infection, that adding ribavirin to pegylated interferon would improve these already high SVR rates. In addition, it would expose patients to an increased risk of anemia and their offspring to congenital abnormalities. In Egypt, where the cost of drugs is a major issue, delaying treatment to allow SVC and using only 12 weeks of pegylated interferon could preserve much-needed resources. There are, however, two caveats related to this strategy: patients may be lost to follow-up if not immediately treated, and the fact that some patients have an atypical pattern of viremia, with transient non detection of viremia in the first six months after onset of symptoms. In this study, 18 of the 66 patients who did not clear the virus spontaneously had transient undetectable viremia during the first six months after onset of symptoms. It is therefore crucial to repeat the HCV PCR during the first six months of follow-up to confirm viral clearance in patients who turn negative at one examination.

In conclusion, this study has documented the high spontaneous clearance rate of symptomatic acute hepatitis C, and shown that 80% of SVC had taken place three months after onset of symptoms. In patients who had not spontaneously cleared the virus by that time, SVR could be obtained in 88.2% with a short 12-week monotherapy with pegylated interferon alfa-2a.
